# Editorial: The dark and the light side of gaming, volume II

**DOI:** 10.3389/fpsyg.2026.1819192

**Published:** 2026-04-13

**Authors:** Teresa de la Hera, Felix Reer, Marko Siitonen

**Affiliations:** 1 Department of Media and Communication, Erasmus University Rotterdam, Rotterdam, Netherlands; 2 Department of Communication, University of Münster, Münster, Germany; 3 Department of Language and Communication Studies, University of Jyväskylä, Jyväskylä, Finland

**Keywords:** digital games, game communities, game effects, game studies, media effects

The academic debate on the effects of digital games has been present throughout the entire history of game studies. It is a reflection of the debates present in our society since the introduction of the first affordable personal computers in the 1980s (Reer et al., 2024), and can also be seen as a continuation of the scholarship into media effects that emerged from the early 20th century onward (see e.g. Valkenburg et al., 2016). Many early takes on the effects of digital games approached their topic from a *technological determinism* perspective (Marshall McLuhan, 1964), in which digital games were studied as the determinants of user behavior, disregarding players attitudes, interaction and context as part of their effects. These studies focused their efforts on shading light on the fears about the potential negative effects that dominated societal debates (e.g., Cooper and Mackie, 1986). However, after their technological domestication, and the further development of the field of game studies, the attention shifted into providing understanding on the positive effects of digital games (e.g., Bediou et al., 2023).

Nowadays, research done across the field of game studies has provided enough knowledge to understand that games and play are multifaceted and complex, and understanding their effects requires putting each study in context and paying attention to concrete aspects of games, players and society.

This Research Topic is the second volume of this Research Topic with which we aim to further expand the awareness about the complexity of the effects of digital games, as well as the broad spectrum of both their potential positive and negative effects. The first Research Topic (Reer et al., 2024) included 13 articles. The current Research Topic adds to that with a total of 10 papers presented in three sections discussing three sides of digital games effects: the light, the dark and the gray area between the two extremes.

## The light side

This section includes five papers that explore the potential positive impacts or applications of digital gaming, providing new insights on the light side of digital games and play.

The study by Cahill et al. explores players' motivations to use games as tools for coping and emotional regulation. Their survey study makes use of the twin theoretical lenses of mood management and stress response theory, to explore this topic. The authors highlight that their results provide clear evidence that immersion and motivation are key factors to understand the role of games as tools used by players for emotional regulation and coping with stress. In their paper, concrete mediating factors such as narrative involvement and social interaction are discussed in relation to this topic.

Another positive application of gaming that has been explored by several authors in the past decade is the role of games to foster intergenerational communication. Sun et al. contribute to previous studies on this topic by exploring how metaphorical life games can be used to facilitate intergenerational communication between teenagers and adults. The authors found that the life review dynamic part of metaphorical life games facilitates intergenerational communication by: (1) providing a communication climate; (2) fostering symmetrical interaction among different generations and (3) facilitating metaphorical expression.

Daneels et al. study aims to understand how games serve as a way to regulate boredom. By using an online vignette-style survey, they applied the meaning and attentional component model to test how meaningful gaming can be to individuals who experience different types of boredom. They found that three thirds of their respondents tend to regulate boredom by playing digital games. Their results also suggest that games offering hedonic experiences are selected over games offering eudaimonic experience by both individuals with high and low cognitive resources.

Bouzek et al. explored the role of playful engagement and interactions among game industry professionals during virtual meetings. By conducting 38 semi-structured interviews, the authors collected data of three different types of manifestations of play during these meetings, including the use of avatars and playful backgrounds for self-representations, gameplay during meetings and playful technological experimentation. The findings show that playful engagement is particularly useful during brainstorm oriented meetings, and is most commonly used among indie game studios.

Rüth et al. conducted a mix methods online study to explore if and how digital games can be used as a tool in psychoeducation aimed at destigmatizing depression. The authors made use of an intervention in which participants were requested to watch gaming videos of a digital game about depression and write down aspects of the videos that left a lasting impression on them. Their findings suggest that this method is effective in supporting depression destigmatization.

## The dark side

Offering a counterpoint to the previous section, this section includes three articles that explore the possible negative effects of digital games and play.

A topic that is currently central in academic studies about the dark side of playing is the relationship between online gaming and extremism. Greipl et al. explore this topic by studying the relationship between gameplay motivations and indicators of radical attitudes and conspiracy beliefs. The main finding of this paper is that the authors were not able to find any strong evidence of gaming as a straightforward driver of extremism. On the contrary, gaming in relation to extremism and radicalization should be studied as a complex context-dependent space that can both facilitate but also prevent radicalization subject to the individual circumstances of the player.

Newhouse and Kowert's study also provides insights into the relationship between gaming and extremism. In concrete the authors dive into extremism identity creation practices on Steam. This study provides evidence on how Steam community features are used by big groups to share hateful and violent content, promoting collective mobilization and radicalization. The authors conclude this study by reflecting on how these practices become a threat to gaming communities and individuals who use Steam in connection to their gaming activities.

Another topic that has gained attention since Esports became mainstream is the wellbeing of Esport players. Kang and Kim contribute to this issue with a study on the factors influencing the career longevity of Esport players. The authors conclude that current practices in the industry, including exploitative contractual practices and intense training schedules are determining factors for short career paths and burnout experiences.

## The gray side

The final section comprises two papers that explore the effects of games from a neutral perspective, that can fall into both the light or dark side of gaming, depending on the application.

In relation to the study of the balance between both the dark and the light side of gaming, Aleksić et al. have analyzed the in-game posters in the *Fallout* game series to identify how these portray ideologies and conflicts. In the study they combined theory from the field of propaganda studies with the support of a multimodal framework for the analysis. Their results show that there is a prevalence of text-dominant posters in which metonymy is the most frequent figure of speech used.

Another study conducted by Hébert-Ratté et al. dives into the characteristics of expert female gamers. The results of their survey shows that expert female games show higher gaming involvement than non-expert female players. Furthermore, these players also seem to express having more interest in social, competition, skill development and recreation game-related aspects than casual female players.

It is with this gray area that we close the two-part Research Topic. Over the course of editing the 24 articles, we often thought about the dichotomy that we initially chose for the title. We fully recognize that dichotomies such as dark/light or positive/negative always over-simplify the complex nature of reality. At the same time they do have their uses, and we fully expect scholarship into the effects of digital games and play to continue utilizing them. For now, though, we wish to remind the reader that many of the articles published over the two Research Topics highlighted the complicated and even unpredictable ways in which digital gaming can be interwoven into our lives. We also encourage scholars to continue on their quest of teasing out relationships and effects despite the potential challenges. Games and play matter, and it is up to research to show how exactly.
